# Comparison of the Effectiveness of a Novel Matrix-Modified Bovine Collagen Membrane With a Conventional Bovine Collagen Membrane for Oral Mucosal Defects: A Single-Center Study

**DOI:** 10.7759/cureus.53696

**Published:** 2024-02-06

**Authors:** Kalyani P, Kathiravan Selvarasu, Karthikeyan Murthykumar, Murugesan Krishnan, Santhosh P Kumar, Saravanan Lakshmanan

**Affiliations:** 1 Department of Oral and Maxillofacial Surgery, Saveetha Dental College and Hospitals, Saveetha Institute of Medical and Technical Sciences, Saveetha University, Chennai, IND; 2 Department of Periodontics, Saveetha Dental College and Hospitals, Saveetha Institute of Medical and Technical Sciences, Saveetha University, Chennai, IND

**Keywords:** innovative practice, novel technique, dressing, granulation, epithelialization, bovine, collagen

## Abstract

Background

Surgical procedures such as excision of a growth or lesion lead to soft tissue or oral mucosal defects. These defects require a proper surgical dressing to promote better wound healing and to avoid infection and scarring. A collagen membrane is one of the most commonly used surgical dressings because of its ease of adaptability to defects and its inherent ability to promote epithelialization and inhibition of pain through the indirect mechanism of preventing infection of the surgical site. Collagen also serves as a reservoir of regenerative factors. The regenerative potential increases as porosity decreases. The novel bovine-derived collagen membrane used in this current study has an average porosity of 20 microns which increases the availability of regenerative factors.

Objective

The aim of this study was to compare the effectiveness between a novel matrix-modified bovine collagen membrane (SurgiColl) and a conventional bovine collagen membrane for promoting wound healing for oral mucosal or soft tissue defects.

Materials and methods

This clinical trial was conducted in the Department of Oral and Maxillofacial Surgery, Saveetha Dental College and Hospital. The sample size of the study was 20, divided into two groups: novel bovine collagen (Surgicoll-Mesh) (Group 1) and conventional bovine collagen (Group 2) with 10 participants in each group. The randomization process was adopted. The parameters assessed were epithelialization, granulation, and wound contraction at the end of two weeks. All the parameters were assessed using a standardized visual assessment scale. Statistical analysis was done using IBM SPSS Statistics for Windows, Version 23.0 (Released 2015; IBM Corp., Armonk, New York, United States), and an independent sample t-test was done at 95% confidence interval. A p-value of less than 0.05 was considered statistically significant.

Results

The difference in epithelialization between the two groups was statistically significant with a p-value of 0.015 (<0.05). The difference in granulation tissue formation between the two groups was statistically significant with a p-value of 0.015 (<0.05). The difference in wound contraction at the end of two weeks between the two groups was also statistically significant with a p-value of 0.005 (<0.05). Group 1 showed superior results compared to Group 2 for all the outcomes assessed.

Conclusion

The novel bovine-derived collagen membrane (SurgiColl-Mesh) was superior in its properties of wound healing for oral mucosal or soft tissue defects than the conventional bovine collagen membrane.

## Introduction

The surgical procedures involving the oral mucosa or soft tissues result in defects that are quite challenging to reconstruct. These defects might be small in their size resulting from excisional biopsy procedures or might be larger defects caused due to wide local excision resection. Such defects, if left without any surgical dressing, will serve as a nidus for infection and will also undergo ulceration due to exposure of the oral cavity to a variety of irritants. This will, in turn, impede the process of wound healing, resulting in scarring, and nonhealing wounds. Furthermore, these wounds will also be a constant source of pain and irritation to the patient. Hence, it becomes essential to provide an appropriate surgical dressing for such defects [1].

The properties of an ideal wound dressing include the promotion of hemostasis; the ability to prevent infection, reduce pain, granulation tissue formation, aid in rapid reepithelialization, and reduce contracture; and the ability to avoid or reduce donor site morbidity [1]. Considering all these factors, a variety of surgical dressings have evolved over the years for use in the oral and maxillofacial region. These include the collagen membranes, buccal fat pads, mucosal grafts, and skin grafts [2]. Though skin grafts provide excellent coverage, they have the disadvantages of the presence of adnexal tissue and donor site morbidity. The collagen membrane has the action of activating platelets, causes platelet aggregation over the raw wound, and strengthens the clot. Collagen also causes chemotaxis, cell aggregation and adhesion, and release reaction of platelets [3]. It has a chemotactic effect on endothelial cells and fibroblasts due to this inflammation, and pain is significantly reduced. The collagen membrane is also easily adapted to the contour of defects and is robust enough to bear the masticatory stress [4].

The bovine collagen membrane is an easily available preparation that also eliminates the donor site morbidity. The conventional collagen membranes of bovine source are usually a product of lyophilization, yielding a random structural configuration, with an average porosity greater than 800 microns. This makes the product inconducive for cell infiltration. Previous studies have assessed the versatility in the use of a collagen membrane in oral mucosal defects [2,3]. However, there are no studies comparing two collagen membranes of different constitution and structure. In the current study, a novel matrix-modified bovine collagen membrane (SurgiColl-Mesh) was used, and the outcomes were compared with that of a bovine collagen membrane. This novel collagen is a product of sediment preparation that yields native parallel fibers with an average porosity of 20 microns that attracts more cells and increases the penetration of growth and regenerative factors. This in turn has the potential to increase the rate and the quality of healing. 

The aim of this study was to compare the effectiveness between the novel matrix-modified bovine collagen membrane (SurgiColl-Mesh) and the conventional bovine collagen membrane in promoting wound healing for oral mucosal defects.

## Materials and methods

Study setting

This study was conducted in Saveetha Dental College and Hospitals from April 2022 till March 2023. Informed consent was obtained from all the participants of the study. Institutional Human Ethical Committee, Saveetha Dental College issued approval (IHEC/SDC/OMFS-2102/22/184) for the study. The study was registered under the Clinical Trial Registry of India (CTRI)-REF/2023/12/076231.

Study design 

This was a phase 1 randomized controlled clinical trial. Patients undergoing a wide local excision of benign neoplasms involving the oral mucosa leading to mucosal defects and patients with oronasal fistula (oro-mucosal defects of similar nature) were included in this study. The eligibility criteria included patients in the age group of 20-60 years. Patients presenting with systemic infections, immunocompromised status, and those who were irradiated were excluded from the study. 

Study groups and sampling 

**Figure 1:**
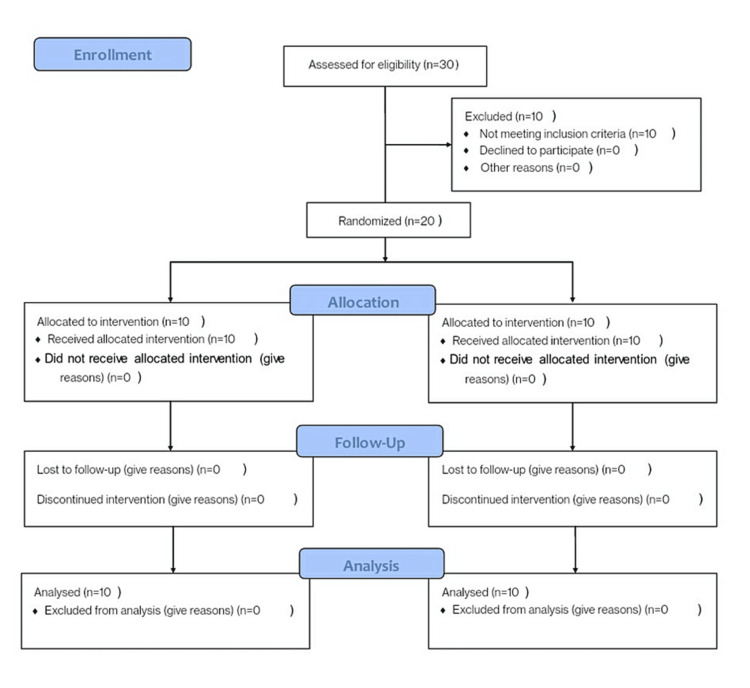
CONSORT flowchart CONSORT: Consolidated Standards of Reporting Trials

The total sample size of the study was determined to be 20 using G-power statistics with a confidence interval set at 85%. The samples were divided into two groups. Randomization was done based on a computer-generated number sequence. Computer-generated random sequence numbers were kept in the sealed opaque envelope for allocation in the two groups. This was a double-blinded trial. The patients and the assessor were blinded. The Consolidated Standards of Reporting Trials (CONSORT) guidelines were followed for the reporting of the trial results (Figure 1).

**Figure 2:**
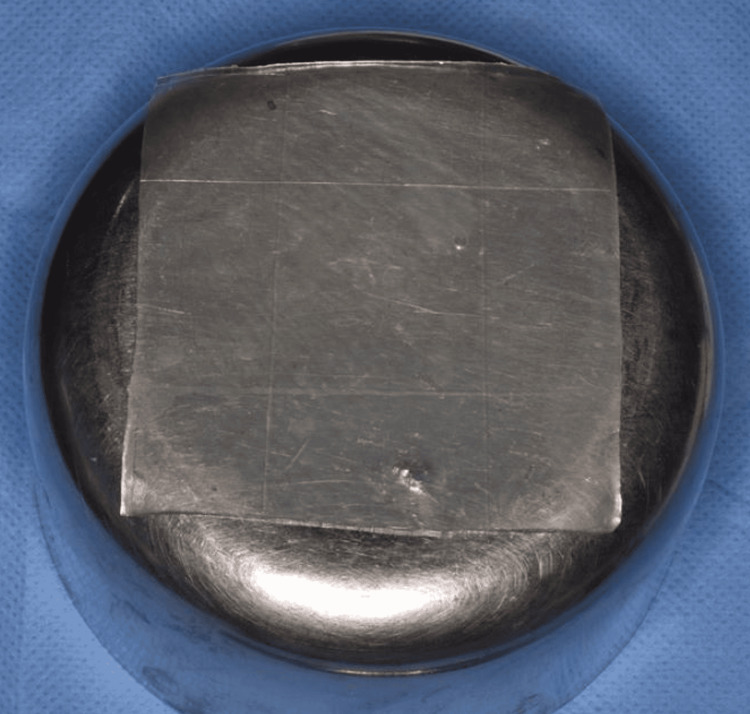
Novel matrix-modified bovine-derived collagen (SurgiColl-Mesh)

In all Group 1 patients, a novel matrix-modified bovine collagen membrane (SurgiColl-Mesh) was placed. It is an implantable, bioresorbable, tissue-regenerative, sterile type I collagen matrix material as shown in Figure 2. In Group 2 patients, a conventional bovine collagen membrane was placed. The sample size was 10 per group.

Surgical procedure

**Figure 3:**
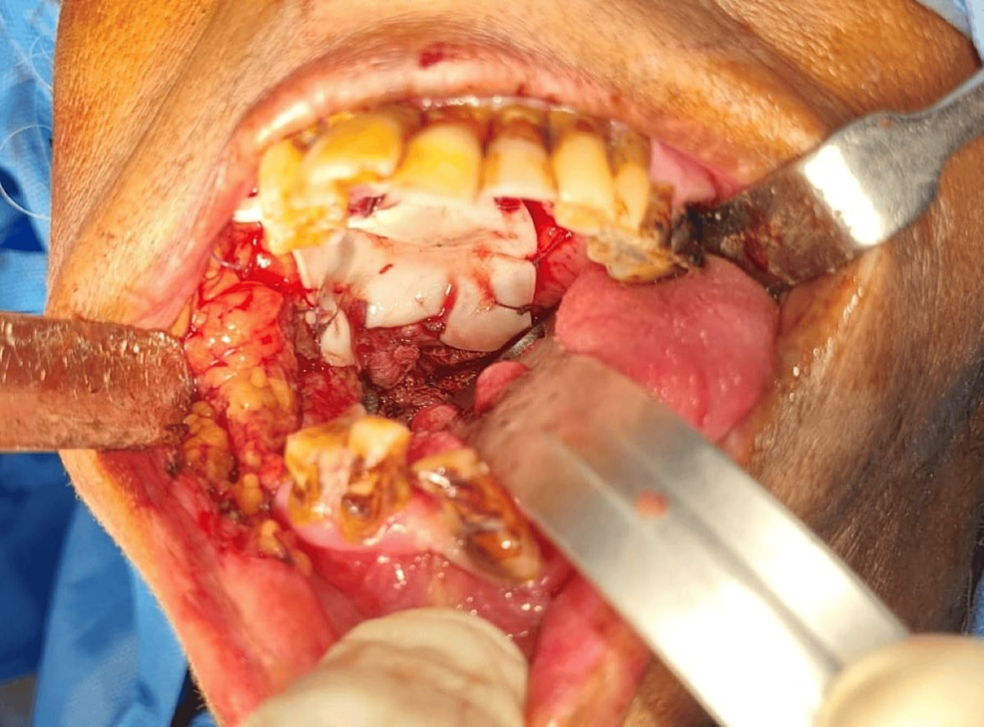
Partial maxillectomy defect closed with SurgiColl

**Figure 4:**
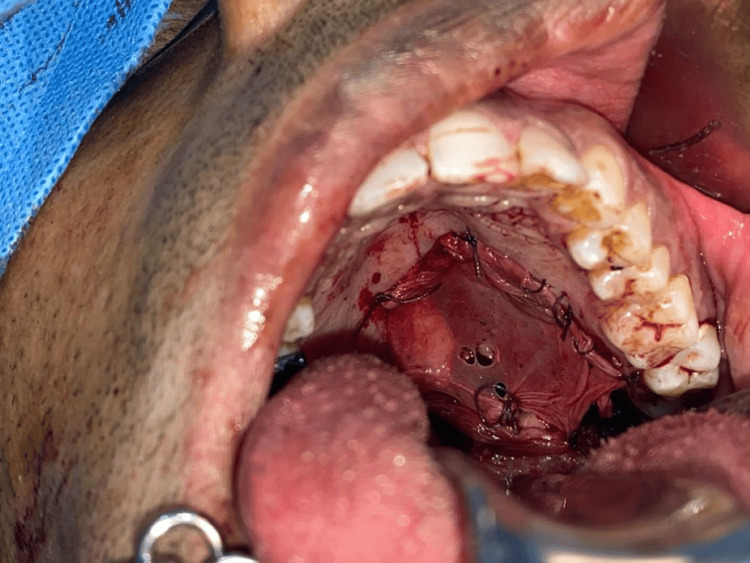
Partial maxillectomy defect closed with conventional collagen

All the cases were performed under general anesthesia. The sample consisted of defects after surgical excision of palatal tumors, closure of oroantral fistulas, in oral submucous fibrosis and in post resection soft tissue defects such as partial glossectomy. The maximum size of the defect observed of all the samples was a 8 x 8 cm defect. After the excision, sufficient hemostasis was obtained. Collagen membrane was adapted to the surgical site and sutured using 3-0 polyglactin sutures in all the cases. Figure 3 depicts a case of partial maxillectomy where primary closure was done with SurgiColl. Figure 4 depicts a case of partial maxillectomy where primary closure was done with conventional collagen. All the patients were under intravenous amoxicillin antibiotic coverage till the third postoperative day. Liquid diet was maintained for the first 24 hours, followed by soft diet till the 7th postoperative day.

Parameters assessed

The presence of granulation tissue was assessed at the end of two weeks using the following scale: 2, GOOD (entire wound); 1, FAIR (nearly the entire wound); and 0, POOR (inadequate). Epithelialization was observed at the end of two weeks: 2, GOOD (entire wound); 1, FAIR (nearly the entire wound); and 0, POOR (inadequate). Contracture of the wound at the end of two weeks was recorded as follows: 2, GOOD (<25%); 1, FAIR (25 to 50%); 0, POOR (severe, i.e., >50%). 

Statistical analysis

The observations were tabulated and analyzed using IBM SPSS Statistics for Windows, Version 2.3 (Released 2015; IBM Corp., Armonk, New York, United States). Normality of the data was checked with the Shapiro-Wilk test. Independent sample t-test was done to determine the statistical significance of the results with a p-value less than 0.05 considered as statistically significant, and bar graphs were plotted.

## Results

The valid sample size of the study was N = 20. The sample size in each group was 10 with Group 1 as a novel bovine collagen membrane (SurgiColl) and Group 2 as a conventional bovine collagen membrane. 

**Figure 5:**
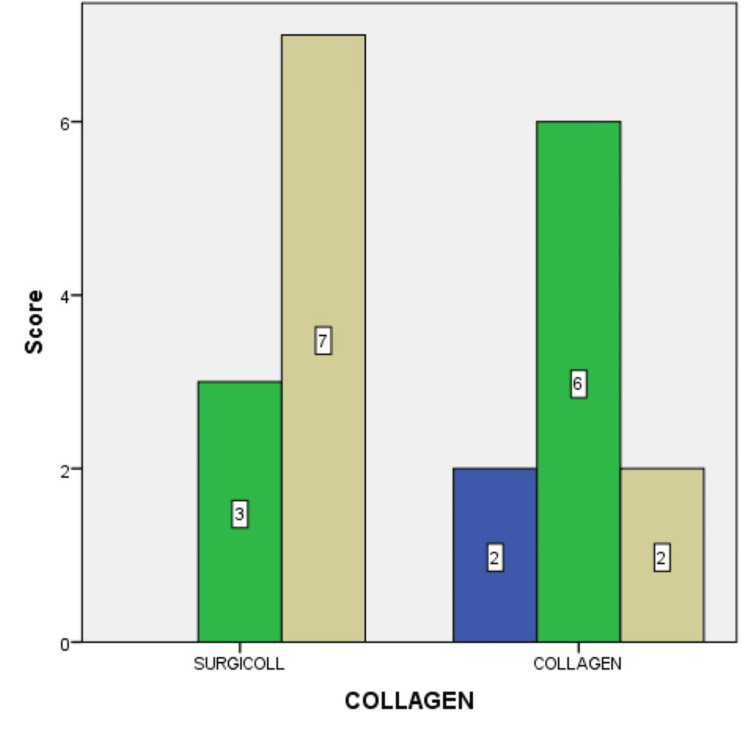
Epithelialization at the end of two weeks between the SurgiColl and the conventional collagen membrane groups Blue: score 0 (POOR); green: score 1 (FAIR); brown: score 2 (GOOD). Bar legends indicate the number of samples.

Figure 5 depicts the difference in epithelialization at the end of two weeks, between the SurgiColl and the conventional collagen membrane groups. Of the overall sample size, 35% exhibited good epithelialization when the SurgiColl membrane was used compared to only 10% of the overall sample size exhibiting the same when the conventional collagen membrane was used.

**Figure 6:**
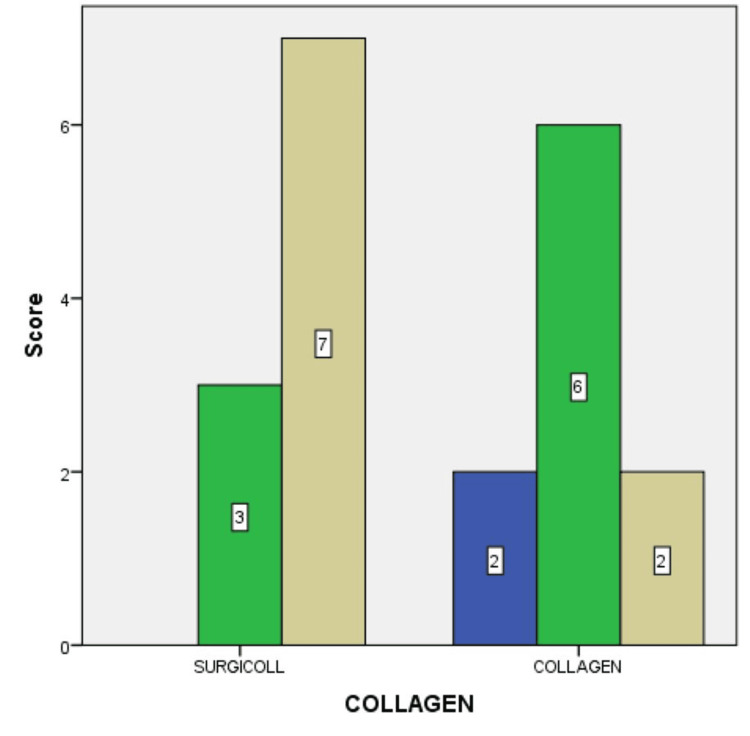
Granulation tissue formation at the end of two weeks between the SurgiColl and the conventional collagen membrane groups Blue: score 0 (POOR); green: score 1 (FAIR); brown: score 2 (GOOD). Bar legends indicate the number of samples.

Figure 6 depicts the difference in granulation tissue formation at the end of two weeks, between the SurgiColl and the conventional collagen membrane groups. Of the overall sample size, 35% exhibited good granulation tissue formation when the SurgiColl membrane was used compared to only 30% of the overall sample size exhibiting the same when the conventional collagen membrane was used. Of the overall sample size, 10% exhibited poor granulation tissue formation when the conventional collagen membrane was used.

**Figure 7:**
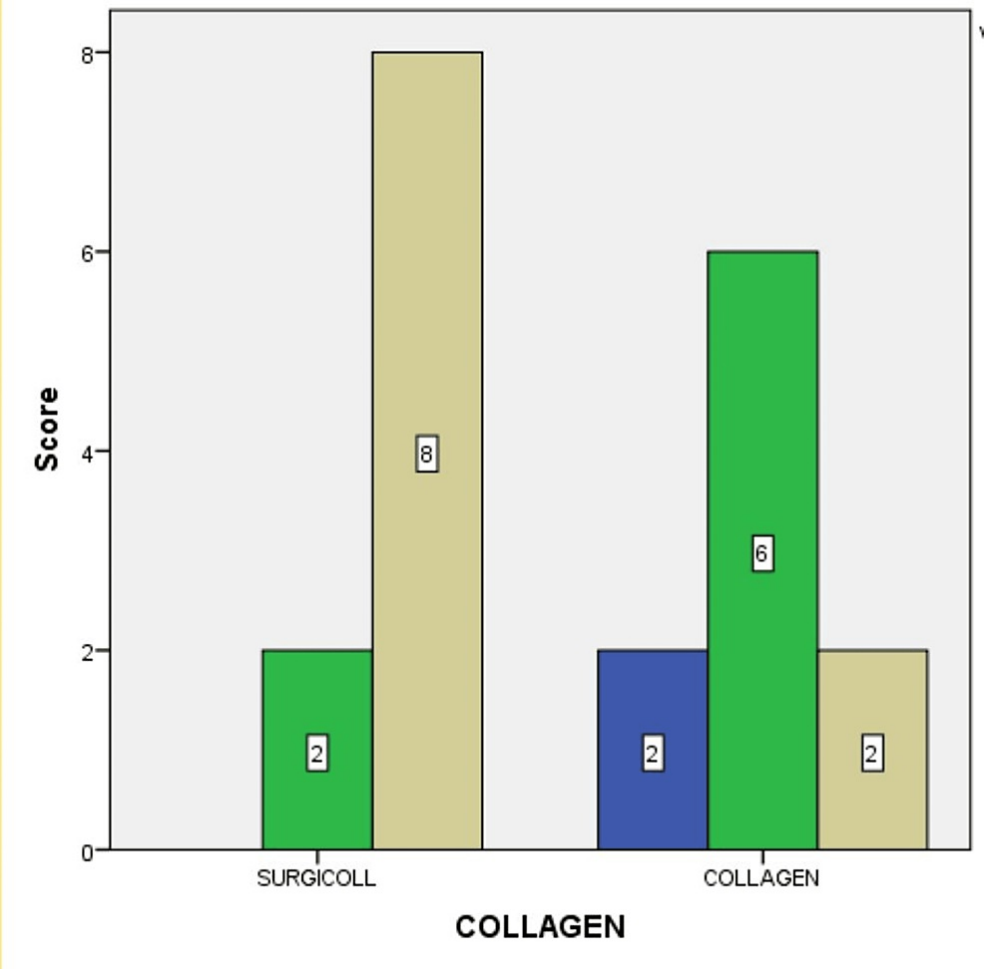
Wound contraction at the end of two weeks between the SurgiColl and the conventional collagen membrane groups Blue: score 0 (POOR); green: score 1 (FAIR); brown: score 2 (GOOD). Bar legends indicate the number of samples.

Figure 7 depicts the difference in wound contraction at the end of two weeks, between the SurgiColl and the conventional collagen membrane groups. Of the overall sample size, 40% exhibited good wound contraction when the SurgiColl membrane was used compared to only 10% of the overall sample size exhibiting the same when the conventional collagen membrane was used. Of the overall sample size, 10% exhibited poor wound contraction when the conventional collagen membrane was used.

**Figure 8:**
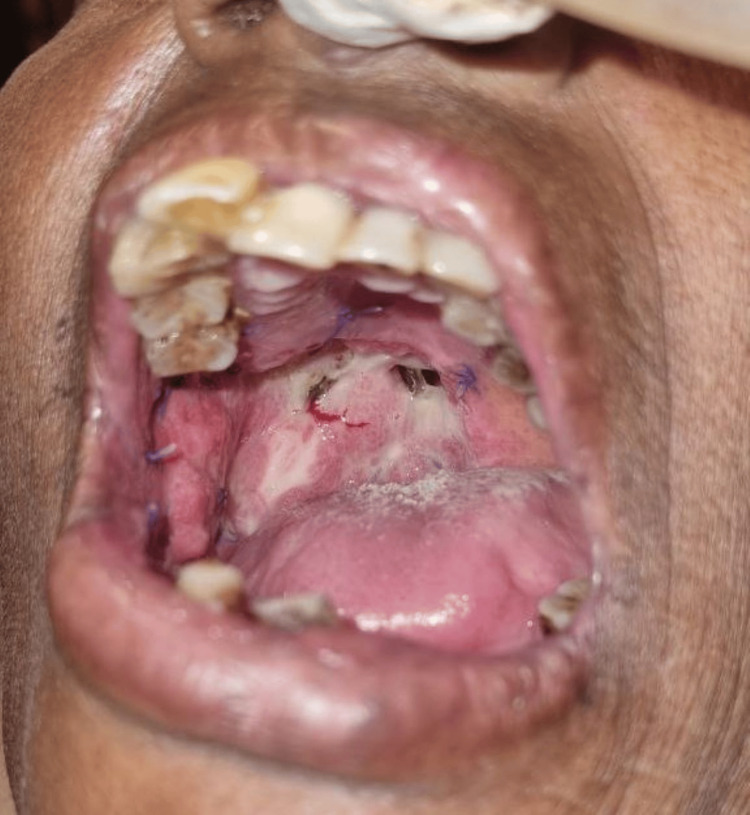
Four weeks postoperative healing of the surgical site covered with a novel matrix-modified bovine-derived collagen membrane (SurgiColl-Mesh)

**Figure 9:**
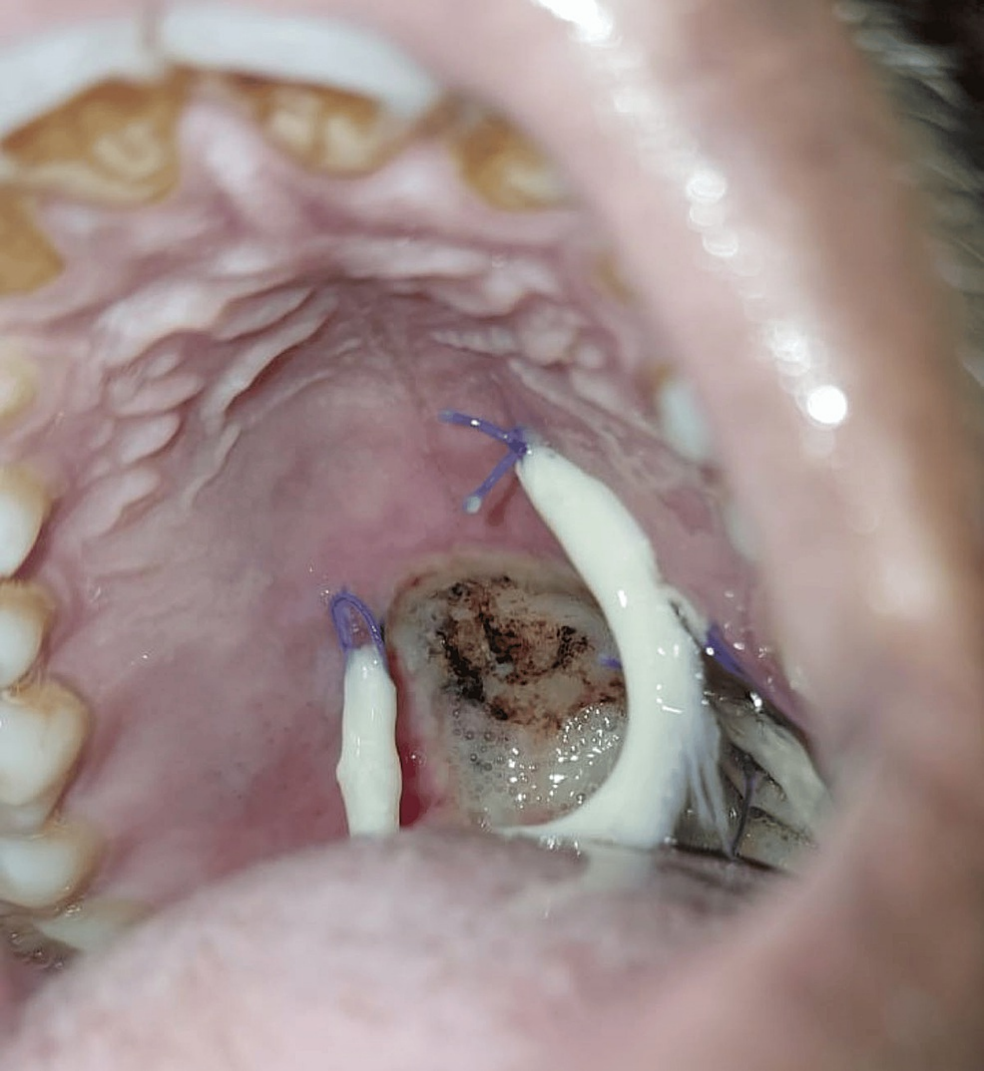
Four weeks postoperative healing of the surgical site covered with a conventional collagen membrane

Four weeks postoperatively, wound healing was evaluated in the SurgiColl (Figure 8) and the conventional collagen membrane groups (Figure 9).

Table 1 shows the demographic details of the study participants.

**Table 1:** Demographic data of the study participants SD: Standard deviation

Demographic Data	Group 1	Group 2
Mean Age in Years 土 SD	42 土 1.5	43 土 2.5
Gender (Male, Female)	6, 4	7, 3
Mean Weight in Kilograms 土 SD	62 土 1.5	64 土 1.3

Table 2 shows the statistical difference between the means of two groups for the parameters assessed using the independent sample t-test. The difference in epithelialization between the two groups was statistically significant with a p-value of 0.015 (<0.05). The difference in granulation tissue formation between the two groups was statistically significant with a p-value of 0.015 (<0.05). The difference in wound contraction at the end of two weeks between the two groups was also statistically significant with a p-value of 0.005 (<0.05). Group 1 showed superior results compared to Group 2 for all the outcomes assessed.

**Table 2:** Statistical assessment of the outcomes between the two study groups *Statistically significant, independent sample t-test

S. No	Outcomes	Groups	Mean 土 Standard Deviation	p-value
1	Epithelialization	Group 1	1.7 土 0.48	0.015*
		Group 2	1.0 土 0.67	
2	Granulation	Group 1	1.7 土 0.48	0.015*
		Group 2	1.0 土 0.67	
3	Wound Contraction	Group 1	1.8 土 0.42	0.005*
		Group 2	1.0 土 0.67	

Outcome parameters like epithelialization, granulation, and wound contraction between the two study groups were assessed at two weeks postoperatively. However, postoperatively patients were being followed up periodically and wound healing was satisfactory and uneventful at the six-month postoperative period.

## Discussion

Epithelialization and granulation tissue formation are the two main processes by which raw wounds in the oral cavity, in particular the oral mucosa exhibit healing [3]. However, during the course of this healing process, the oral cavity is susceptible to a range of forces caused by mastication, deglutition, and occlusion as well. The environment is also exposed to food substances and is constantly under the coverage of saliva, making it susceptible to the action of microflora. All these factors might interfere with the healing of such raw mucosal wounds. This necessitates the need for a proper wound cover or wound dressing for the healing to occur amicably. According to the basic surgical principles, this wound dressing is necessary to prevent infection, scarring, and nonhealing [4]. Biological wounds are the ideal wound dressing as they create a nearly ideal physiologic environment for the wound surface to heal [4].

A collagen membrane is an easily available, ready-to-use, biological skin or mucosal substitute that has been long used even as a culture medium for the culture of cells such as osteoblasts. According to existing literature evidence, collagen affects wound healing in all its stages [5]. Collagen acts as a scaffold and facilitates the migration and infiltration of fibroblasts and lymphocytes. It also inhibits the action of matrix metalloproteinases, further increasing the deposition of collagen by fibroblasts [1,3,5]. This property was also evident in the current study, with the novel bovine-derived collagen membrane exhibiting better wound contraction (wound healing) than the conventional collagen membranes. The use of the collagen membranes also facilitates better epithelialization. This can be attributed to the property of the collagen membranes to stabilize the blood clot, promoting faster epithelialization and reducing scarring.

According to Sowjanya et al. [2], wound dressing provided by collagen acts as a coverage for free nerve endings, thereby reducing the degree of postoperative pain in the surgical site. This is explained by the fact that collagen is a substitute for extracellular matrix and is chemotactic to cells such as fibroblasts. This reduces the overall degree of the inflammatory process, reducing the pain and burning sensation. Kumar et al. [6] in their study on the use of a collagen membrane in oral submucous fibrosis pointed out that it minimized the pain, edema, and infection, and the wound texture was restored to normal in one month. Jasthi et al. have recorded the biodegradability of the collagen membrane and have stated that most of the collagen membrane undergoes lysis by the end of seven days [7]. Hence, in our current study, a fixed time period of two weeks was taken to record all the parameters associated with wound healing, namely, epithelialization, granulation tissue formation, and wound contraction. 

However, according to Mahajan et al. [8], platelet-rich fibrin was still superior to the collagen membrane, as it produces lesser postoperative pain, comparatively faster healing and fewer postoperative complications than the collagen membrane after surgical excision of oral mucosal lesions. Bovine collagen membranes are one of the commonly used derived collagen materials. The porcine collagen membrane has also been used in the literature in a study by Herford et al. [9], with the graft exhibiting an overall shrinkage of 14%. The study does not report any comparison between porcine and bovine collagen matrices in the same regard. Thoma et al. [10] have recorded faster rates of re-epithelialization in oral mucosal wounds in sites covered with collagen matrix than the sites that were left uncovered. Buccal fat pad is another common graft material used particularly in cases of oral submucous fibrosis. Kothari et al. [11] have conducted a study comparing the effectiveness of the buccal fat pad and the collagen membrane in the surgical management of oral submucous fibrosis. They have pointed out that the use of the collagen membrane aided in better postoperative mouth opening, faster epithelization rate, and better wound contracture, though the results were not statistically significant. Blagushina et al. have reported similar results in their study [12].

In the current study, two different types of collagen membranes were compared. The results show that the novel bovine-derived collagen membrane, SurgiColl, was superior in the properties of wound healing, namely, epithelialization, granulation tissue formation, and wound healing than the conventional collagen membrane. There was no significant cost difference between the two types of collagen membranes used in this study. The results were both clinically and statistically significant for all the three evaluated parameters. No adverse reactions were observed in both the study groups which is in accordance with the published literature [13-16].

Limitations of the study

The limitations of the current study are its small sample size. This preliminary original research study gives an insight into the application and use of this novel bovine-derived collagen membrane in the field of oral and maxillofacial surgery. Further multicenter research with larger sample sizes and longer follow-up periods is warranted to validate and generalize these findings. Further, the operator comfort in using the novel matrix-modified collagen membrane must be assessed in the future studies.

## Conclusions

It can be concluded from our study that the novel matrix-modified bovine-derived collagen membrane was more effective in promoting wound healing in oral soft tissue or mucosal defects compared to conventional bovine collagen membranes. This can be attributed to the increased regenerative potential of the novel bovine-derived collagen membrane. Future studies must focus on measuring the long-term effects of the collagen membrane on wound healing.
